# Building Community Space for Supporting Residents Living With Dementia in a Housing Complex District in Tokyo, Japan

**DOI:** 10.1093/geroni/igaa057.182

**Published:** 2020-12-16

**Authors:** Mika Sugiyama, Tsuyoshi Okamura, Fumiko Miyamae, Ayako Edahiro, Madoka Ogawa, Hiroki Inagaki, Chiaki Ura, Shuichi Awata

**Affiliations:** 1 Tokyo Metropolitan Institute of Gerontology, Tokyo, Tokyo, Japan; 2 Tokyo Metropolitan Institute of Gerontology, itabasi-ku, Tokyo, Japan; 3 Tokyo Metropolitan Institute of Gerontology, Itabashi-ku, Tokyo, Japan

## Abstract

It is estimated that by 2025 the number of people with dementia will reach around 600 thousand, approximately one out of five in the older population in Tokyo, Japan. At the same time, the number of older people living in a single, couple household is expected to increase. We built a community space for older people in the largest housing complex district in Tokyo, and with the goal of creating a dementia friendly community (DFCs). In this study, we used the community-based participatory research approach to create a model of an inclusive community space with a human-rights-based approach, which is embodied in the PANEL framework by the Alzheimer Scotland organization. The community space where everyone, regardless of with or without dementia, can freely spend their time, and seek consultation on healthcare and older care. It also serves as a Dementia Café, where people with dementia can get together and chat. Places open 3 days a week. Those users can casually seek consultation by physicians, health nurses and psychologists. From April 1, 2017 to March 30, 2018, the average number of visitors was 11.6. Number of consultation was 182 times (female 81.3%, 80s’ =31.3%; 70s’ =23.1%). Historically, service delivery for the people with dementia was hospital-based in japan, but our community space established a new method to provide consultation to people with dementia, from a professional perspective, and to cooperate with appropriate social resources and related organizations as needed.

